# Shrimp allergic patients are at risk when eating mealworm proteins

**DOI:** 10.1186/2045-7022-5-S3-P77

**Published:** 2015-03-30

**Authors:** Henrike Broekman, André Knulst, Stans Den Hartog Jager, Marco Gaspari, Govardus De Jong, Geert Houben, Kitty Verhoeckx

**Affiliations:** 1 UMC Utrecht, Utrecht, The Netherlands; 2 Università Catanzaro, Catanzaro, Italy; 3 TNO, Zeist, The Netherlands

## 

Due to the imminent growth of the world population, shortage of protein sources for human consumption will arise in the near future. Alternative and sustainable protein sources like insects, such as mealworm, are now being explored for the production of food and feed. Before novel products can be launched on the market the assessment of food safety is vital. One of the key aspects of food safety is the risk of the development of food allergy. Food allergies specifically have a significant impact on the quality of life of allergic patients and their daily functioning, and they may even be life-threatening. TNO together with the University Medical Center Utrecht (UMCU) developed a risk assessment strategy to assess the allergenicity of novel proteins, in accordance to the European Food Safety Authority (EFSA) guidelines to assess the safety of Genetic modified organisms. Using this strategy the safety of mealworm (Tenebrio molitor L.) proteins for use in human consumption was assessed. In this study, 15 shrimp allergic patients were tested on their allergic reaction to mealworm proteins. For this purpose different in vivo (skin prick tests) and in vitro tests (immune blot and basophil activation) were used. In all tests a positive reaction to mealworm extracts was seen. This indicates that shrimp allergic patients are at risk for developing allergic reactions when consuming food containing mealworm proteins. Double blind placebo controlled food challenge will have to confirm this in the coming months.

